# Engineering *Escherichia coli* for highly efficient production of lacto-*N*-triose II from *N*-acetylglucosamine, the monomer of chitin

**DOI:** 10.1186/s13068-021-02050-5

**Published:** 2021-10-08

**Authors:** Duoduo Hu, Hao Wu, Yingying Zhu, Wenli Zhang, Wanmeng Mu

**Affiliations:** 1 State Key Laboratory of Food Science and Technology, Jiangnan University, Wuxi, 214122 Jiangsu China; 2 International Joint Laboratory on Food Safety, Jiangnan University, Wuxi, 214122 Jiangsu China

**Keywords:** Lacto-*N*-triose II, *N*-Acetylglucosamine, *Escherichia coli*, Metabolic engineering

## Abstract

**Background:**

Lacto-*N*-triose II (LNT II), an important backbone for the synthesis of different human milk oligosaccharides, such as lacto-*N*-neotetraose and lacto-*N*-tetraose, has recently received significant attention. The production of LNT II from renewable carbon sources has attracted worldwide attention from the perspective of sustainable development and green environmental protection.

**Results:**

In this study, we first constructed an engineered *E. coli* cell factory for producing LNT II from *N*-acetylglucosamine (GlcNAc) feedstock, a monomer of chitin, by introducing heterologous β-1,3-acetylglucosaminyltransferase, resulting in a LNT II titer of 0.12 g L^−1^. Then, *lacZ* (lactose hydrolysis) and *nanE* (GlcNAc-6-P epimerization to ManNAc-6-P) were inactivated to further strengthen the synthesis of LNT II, and the titer of LNT II was increased to 0.41 g L^−1^. To increase the supply of UDP-GlcNAc, a precursor of LNT II, related pathway enzymes including GlcNAc-6-P deacetylase, glucosamine synthase, and UDP-*N*-acetylglucosamine pyrophosphorylase, were overexpressed in combination, optimized, and modulated. Finally, a maximum titer of 15.8 g L^−1^ of LNT II was obtained in a 3-L bioreactor with optimal enzyme expression levels and β*-*lactose and GlcNAc feeding strategy.

**Conclusions:**

Metabolic engineering of *E. coli* is an effective strategy for LNT II production from GlcNAc feedstock. The titer of LNT II could be significantly increased by modulating the gene expression strength and blocking the bypass pathway, providing a new utilization for GlcNAc to produce high value-added products.

**Supplementary Information:**

The online version contains supplementary material available at 10.1186/s13068-021-02050-5.

## Background

Human milk oligosaccharides (HMOs) are recently attracting increasing attention, as they have numerous excellent physiological functions in human infants, such as promoting brain development in the newborn [1, 2], improving their intelligence [3], avoiding infection [4, 5], and acting as prebiotics [6]. To date, more than 200 different structures have been found and identified in HMOs, composed of five various monosaccharides including glucose (Glc), galactose (Gal), *N*-acetylglucosamine (GlcNAc), L-fucose (Fuc), and sialic acid (SA) [7]. Lacto-*N*-triose II (LNT II, GlcNAc*β*1-3Gal*β*1-4Glc) is an important precursor serving as a backbone to synthesize different HMOs, such as lacto-*N*-neotetraose (LNnT, Gal*β*1-4GlcNAc*β*1-3Gal*β*1-4Glc) and lacto-*N*-tetraose (LNT, Gal*β*1-3GlcNAc*β*1-3Gal*β*1-4Glc), which itself is generated by the catalytic reaction of β-1,3-acetylglucosaminyltransferase (LgtA) on GlcNAc and β*-*lactose substrate [8]. LNnT and LNT are also important components of HMOs, and they can be further fucosylated or sialylated, thereby generating other HMOs, such as lacto-*N*-fucopentaose II (LNFP-II), S-LNF II, lacto-*N*-neofucopentaoses (LNnFP I), among others [9]. These fucosylated or sialylated HMOs also exhibit superior physiological functions for infant health, such as immunomodulatory property [10], and antiadhesive/antimicrobial activity [11]. Therefore, efficient and large-scale production of LNT II is receiving significant attention in the field of HMOs, as the preparation of LNT II with a high titer facilitates the production of other HMOs.

Traditionally, chemical synthesis of LNT II generally involves multiple steps of activation, protection, glycosylation and deprotection, with low yields [6, 12]. Recently, biotechnological production of LNT II through enzymatic and cell factory methods is receiving great attention. Several studies have been carried out for the synthesis of LNT II in vivo and in vitro [13]. For example, 4.7 g L^−1^ of LNT II was obtained using purified β-*N*-acetylhexosaminidase from *Tyzzerella nexilis* towards *p*NP-NAG and β-lactose as substrates [14] (Table 1). However, the enzyme dosage and the expensive nucleotide donors limit their industrial applications. On the other hand, using glycerol as carbon source and energy, Baumgartner et al. constructed an LNT II-producing strain by metabolically engineering a β-galactosidase-negative *E. coli*(Δ*lacZ*) via chromosomal integration of LgtA, resulting in 2.465 g L^−1^ of LNT II [15]. Similarly, Zhu et al. overexpressed GlmS (glucosamine-6-phosphate synthase), GlmM (glucosamine synthase), GlmU (UDP-*N*-acetylglucosamine pyrophosphorylase), and LgtA in *E. coli* (Δ*nagB*Δ*lacZ*Δ*wecB*), and optimized the gene expression strength and translation rates, forming 46.2 g L^−1^ of LNT II using glycerol and β-lactose as a carbon source and substrate in a 5-L fed-batch fermentation [16]. In contrast to the above, LNT II production using metabolically engineered cells, McArthur et al. prepared LNT II by employing Nahk, GlmU, and LgtA enzymes using GlcNAc substrate through the one-pot multienzyme (OPME) strategy in vitro [9]. Although the conversion efficiency of this OPME is high (97%, LNT II/lac), the higher enzyme production cost and low yields make it unsuitable for large-scale production of LNT II. The microbial hosts show attractive properties of efficient pathways for nucleotide phosphate sugar production, lactose uptake and export of HMOs [17]. Therefore, production of HMOs through cell factories has becoming the most industrially promising method.

**Table 1:** Biotechnological production of LNT II through enzymatic and cell factory approaches

Strategies	Substrates	Purified/integrated/overexpressed enzymes	Production condition	Titer	Reference
Single-enzyme	*p*NP-NAG, β-lactose	Purified β-*N*-acetylhexosaminidase	70 °C, pH 5.0	4.7 g L^−1^	[14]
OPME	GlcNAc, β-lactose	Purified Nahk, GlmU, PpA, LgtA	30 °C, pH 8.0	1.54 g (70 mL reaction mixture)	[9]
OPME	Chitin, β-lactose	PbChi70, HaHex74	40 °C, pH 7.5	8.6 g L^−1^	[8]
*E. coli* (Δ*lacZ*)	Glycerol, β-lactose	Chromosomally integrated LacY and LgtA	30 °C, 90 rpm, shake flask (250 mL)	2.465 g L^−1^	[15]
*E. coli* (Δ*lacZ*Δ*nagB*Δ*wecB*)	Glycerol, β-lactose	Overexpressed LgtA, GlmM, GlmU, GlmS	25 °C, fed-batch (5-L)	46.2 g L^−1^	[16]
*E. coli* (Δ*lacZ*Δ*nanE*)	GlcNAc, β-lactose	Overexpressed NagA, GlmM, GlmU, LgtA	25 °C, fed-batch (3-L)	15.8 g L^−1^	This work

*OPME* one-pot multienzyme

Interestingly, GlcNAc is a good and low-cost substrate material, having been employed for the production of various chemical products, such as *N*-acetylneuraminic acid (Neu5Ac) [18], lipids [19], LNT II, and LNnT [8]. GlcNAc has a monomeric structure of chitin, which is a relatively abundant biomass in nature [20]. Numerous studies have been carried out with the aim to obtain GlcNAc or (GlcNAc)_2_ from chitin, and various technical challenges have been overcome, such as the pretreatment of chitin using mechanical grinding and bacterial fermentation [21], high-pressure homogenization [22], steam-explosion [23], KOH or KOH-urea [24], microwave [25], or ultra-sonication methods [26], followed by the enzymatic hydrolysis process combining the use of chitinase [27] and *N*-acetylhexosaminidase [28], obtaining the GlcNAc or (GlcNAc)_2_ product. Thus, producing high value-added products using the GlcNAc substrate is crucial from the point of view of sustainable development. For example, Neu5Ac was produced from GlcNAc by whole-cell catalysis containing overexpressed *N*-acetylglucosamine 2-epimerase and *N*-acetylneuraminic acid lyase [18]. A three-enzyme synthetic system was also developed for the production of LNT II and LNnT from powdery chitin with a titer of 8.6 and 2.0 g L^−1^, respectively [8]. However, there are few available reports about the production of LNT II using GlcNAc substrate by a metabolically engineered cell factory, even though metabolic engineering is recognized as a powerful strategy to synthesize various HMOs. Therefore, it is necessary to complement this aspect of research to promote the production of value-added products from sustainable biomass feedstock.

The *E. coli* strain cannot synthesize LNT II due to the lack of a naturally endogenous synthesis pathway of LNT II in the cells. However, the genome-scale metabolic network analysis indicates that the *E. coli* strain can naturally synthesize the precursor of LNT II, UDP-GlcNAc, under three consecutive biocatalysis processes via GlmS, GlmM, and GlmU from Fru-6-P, which is an important intermediate of the glycolytic pathway that provides the possibility for constructing the biosynthesis pathway for LNT II production in *E. coli* cells [16]. In this study, the biosynthesis pathway for LNT II production was first redesigned by introducing heterologous LgtA from *Neisseria meningitides* (NmlgtA) in *E. coli*, based on the GlcNAc as a sole carbon source and β*-*lactose as the substrate. In order to avoid the feedback inhibition of GlmS, the *nagA* was heterologously overexpressed in *E. coli* cells for the first time for the production of LNT II. Then, the *lacZ* responsible for lactose hydrolysis and *nanE* responsible for the bypass pathway of GlcNAc-6-P epimerization to ManNAc-6-P were inactivated by attempting to supply the lactose receptor and UDP-GlcNAc donor to sufficiently strengthen the synthesis of LNT II. Besides, to accelerate the supply of UDP-GlcNAc (a precursor of LNT II) from GlcNAc-6-P (a phosphorylation product of GlcNAc), related pathway enzymes including NagA (GlcNAc-6-P deacetylase), GlmM, and GlmU, were combinatorially overexpressed, and their gene expression strengths were modulated by optimizing the plasmid copy numbers. Finally, large-scale production of LNT II using GlcNAc as a sole carbon source and lactose substrate was also performed in a 3-L bioreactor and with a final titer of 15.8 g L^−1^ after fermentation for 76 h, presenting a novel platform to synthesize LNT II from GlcNAc, a monomer product of chitin.

## Results and discussion

### ***Construction of biosynthesis pathway for LNT ***II*** production using GlcNAc feedstock***

The overexpression of heterologous LgtA from *N. meningitides* (NmlgtA) in the *E. coli* strain achieves the generation of LNT II, as this enzyme catalyzes the transfer of the GlcNAc residue of UDP-GlcNAc to lactose, which has been reported by numerous studies [9, 29, 30]. Using this strategy, Zhu et al. successfully constructed the biosynthesis pathway for LNT II production by introducing heterologous NmLgtA in the *E. coli* strain when using glycerol as the carbon source [16]. In their study, glycerol must be converted to Fru-6-P through a series of chemical reactions, after which the generated Fru-6-P is converted to GlcN-6-P by GlmS, an initiating enzyme responsible for catalyzing the key step for LNT II production from Fru-6-P. However, the activity of GlmS is inhibited by its catalytic product GlcN-6-P, thus becoming the rate-limiting step during the LNT II synthesis process [31, 32]. Although studies have been carried out on the directed evolution and mutation of GlmS, the obtained GlmS mutant only serves to alleviate the feedback inhibition and does not completely resolve these inhibition issues [33].

**Fig. 1:**
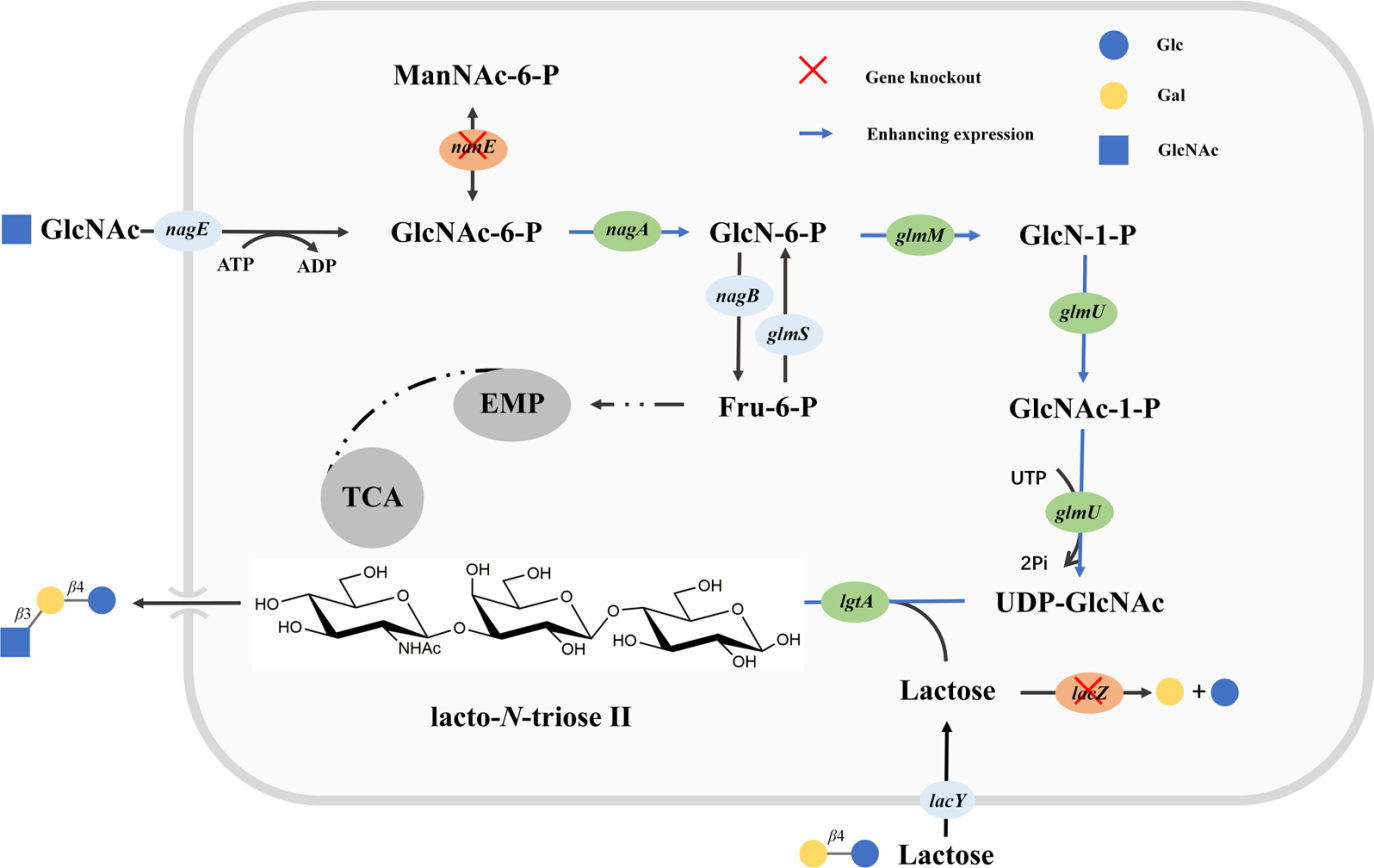
Construction the biosynthesis pathway for LNT II production using GlcNAc substrate in *E. coli*

**Fig. 2:**
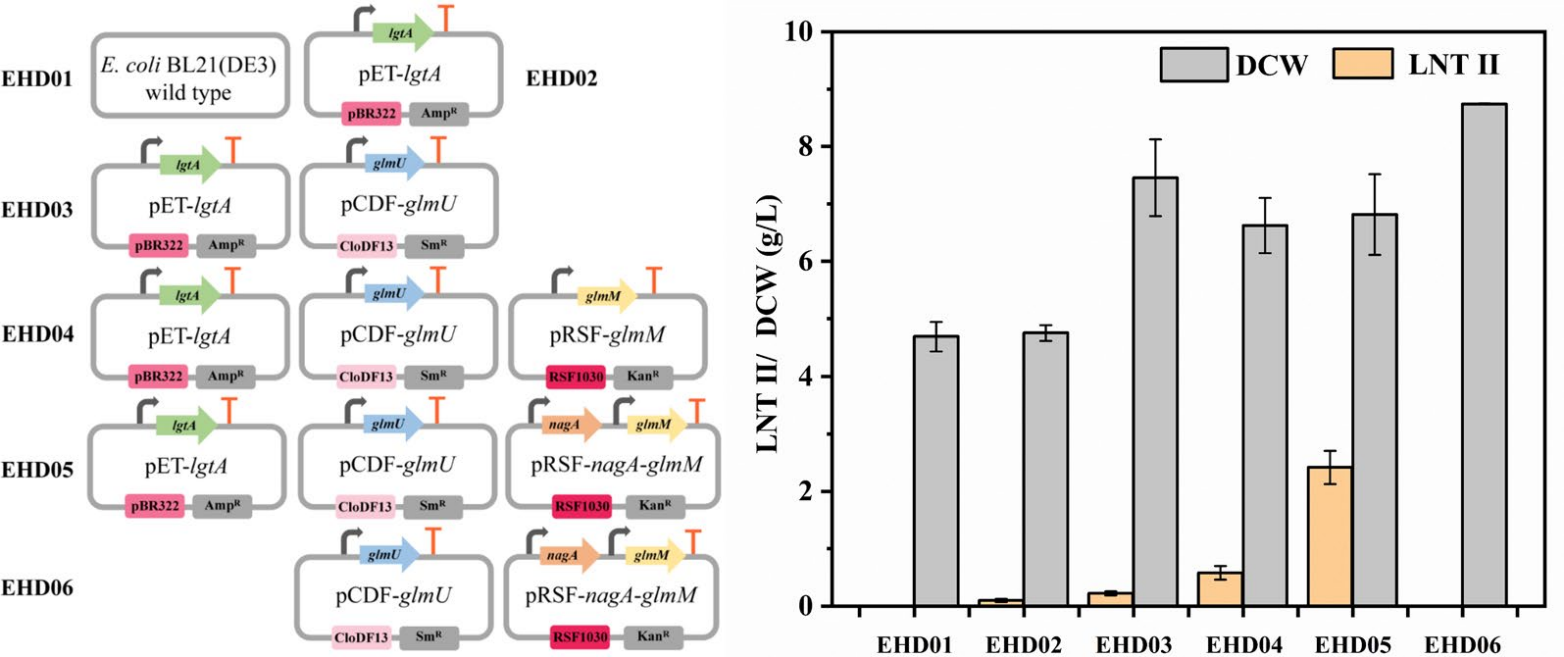
Different overexpression strategies for biosynthesis pathway-related enzymes on the production of LNT II. Left: Amp^R^, ampicillin-resistance gene; Sm^R^, streptomycin-resistance gene; Kan^R^, kanamycin-resistance gene; pBR322, replication origin of pETDuet-1; CloDF13, replication origin of pCDFDuet-1; RSF1030, replication origin of pRSFDuet-1; bent arrows indicate a promoter and T-shaped bars indicate a transcription terminator. Right: the DCW and LNT II of different engineered *E. coli* strains harboring various plasmids. Error bars indicate standard deviations (*n* = 3)

In this study, the metabolic network for LNT II synthesis is redesigned based on the sustainable biomass feedstock GlcNAc. Compared with glycerol substrate, GlcNAc can in theory be rapidly metabolized into GlcN-6-P using endogenous NagE (a GlcNAc-specific transporter that phosphorylates GlcNAc to GlcNAc-6-P) and NagA (a deacetylase that converts GlcNAc-6-P to GlcN-6-P) (Fig. 1). Subsequently, the formed GlcN-6-P can be transformed to UDP-GlcNAc via the catalytic process of GlmM and GlmU. Therefore, we confirmed the possibility of LNT II synthesis by introducing NmlgtA in *E. coli* BL21(DE3) using GlcNAc as a sole carbon source on the defined medium I. It is expected that the engineered strain EHD02 harboring pET-*lgtA* can produce LNT II with an extracellular titer of 0.12 g L^−1^ in the culture broth, when compared with the wild type *E. coli* BL21(DE3) strain EHD01 (no LNT II accumulation) (Fig. 2). We further tested the effect of individual overexpression of GlmU, combinatorial expression of GlmU and GlmM, combinatorial expression of GlmU, GlmM, and NagA, along with NmlgtA in *E. coli* BL21(DE3) on the production of LNT II, generating engineered EHD03, EHD04, and EHD05 strains, respectively (Fig. 2). The LNT II titers of these three engineered strains were 0.24, 0.60, and 2.44 g L^−1^, respectively, with corresponding gains of 100, 400, and 1933% compared to that of EHD02. This suggests that the enhanced combinatorial expression of the three enzymes has a positive effect on the production of LNT II. In contrast, if heterologous NmLgtA is not introduced in the *E. coli* strain, LNT II cannot be produced in the culture broth though the engineered EHD06 strain overexpresses NagA, GlmM, and GlmU enzymes (Fig. 2).

**Fig. 3:**
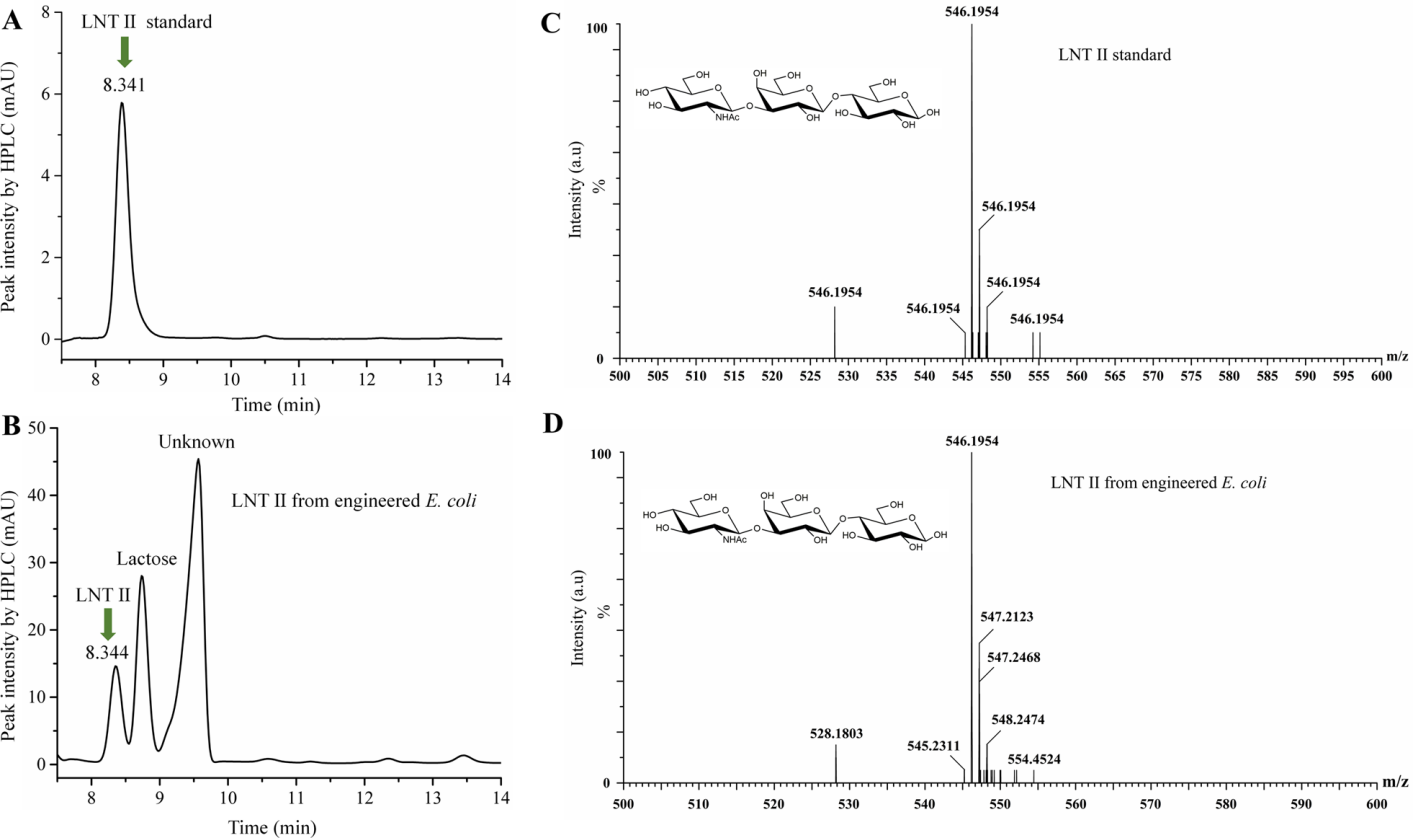
HPLC and UPLC–MS spectrum of LNT II. A and B represent the HPLC profiles of standard LNT II and culture broth from engineered *E. coli* strain, respectively. C and D represent the UPLC–MS profiles of standard LNT II and culture broth from engineered *E. coli* strain, respectively

High-performance liquid chromatography (HPLC) and UPLC–MS were also used to confirm the production of LNT II by the engineered *E. coli* strain. As shown in Fig. 3A, the standard LNT II sample had a retention time of 8.341 min. The culture broth from engineered *E. coli* was also eluted with an identical retention time of 8.344 min (Fig. 3B). The MS information of standard LNT II and culture broth from engineered *E. coli* is displayed in Fig. 3C, D, respectively. Both samples have the same ion fragment of m/z 546.19 under the positive ion mode, indicating that the engineered *E. coli* is capable of producing LNT II when using GlcNAc as the carbon source.

### Effects of inactivating *lacZ* and *nanE* on LNT II production

**Fig. 4:**
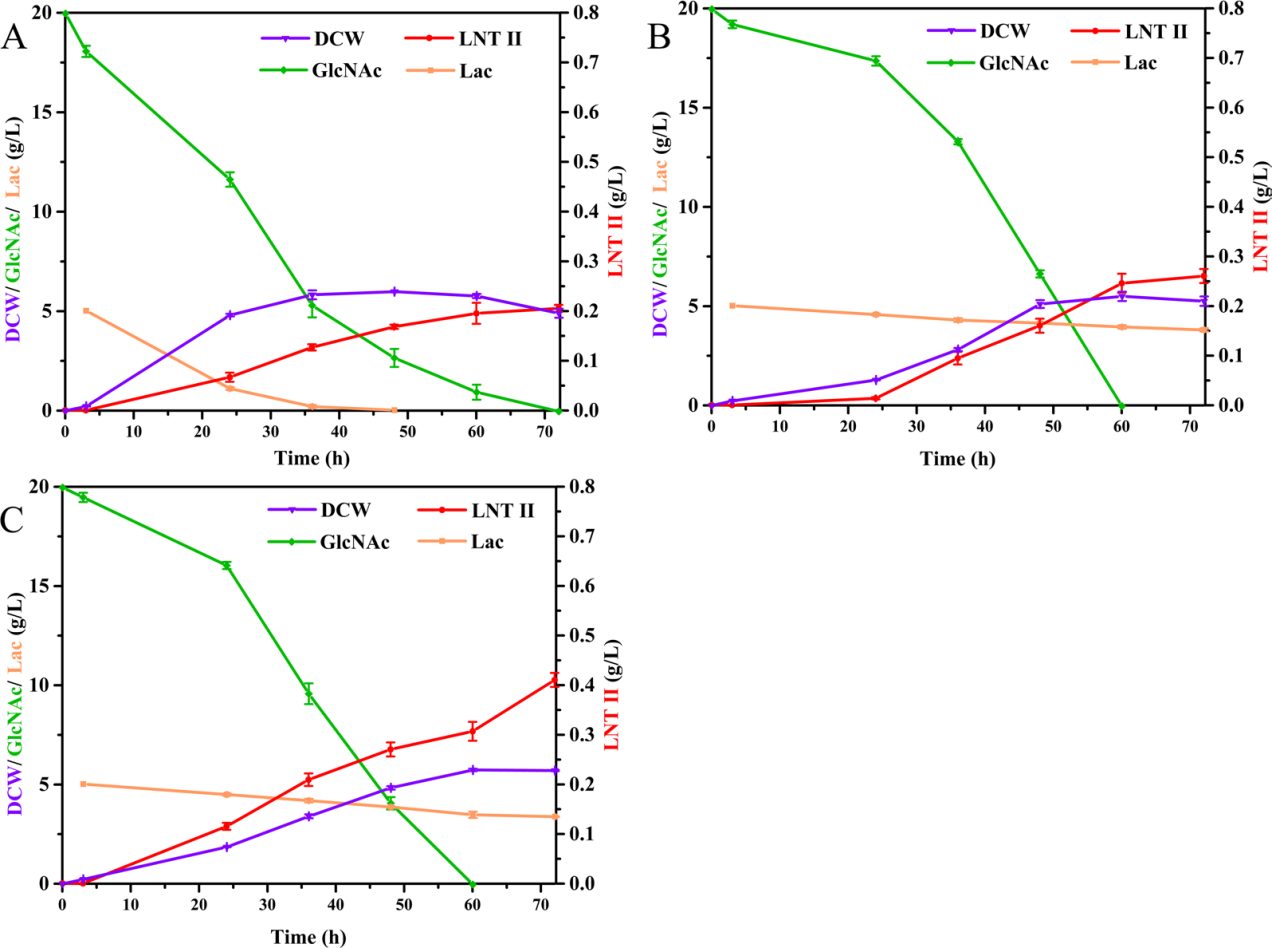
Effects of inactivating *lacZ* and *nanE* on DCW, LNT II titer, residue concentration of GlcNAc and lactose. **A** Engineered *E. coli* BL21(DE3) (EHD07); **B** engineered *E. coli* BL21(DE3) Δ*lacZ* (EHD08); **C** engineered *E. coli* BL21(DE3) Δ*lacZ*Δ*nanE* (EHD24)

The CRISPR–Cas9 technology is a powerful tool to knock-out genes [34]. In this study, CRISPR–Cas9 technology was employed to knock out the genes *lacZ* and *nanE*. Lactose is an important receptor for generating LNT II after transferring GlcNAc residue of UDP-GlcNAc to lactose by LgtA. Thus, sufficient supply and efficient utilization of lactose are essential for LNT II production. However, lactose can be hydrolyzed to Glc and Gal by endogenous β-galactosidase (encoded by *lacZ*) in *E. coli* after entering into cells through permease [35]. Thus, blocking this hydrolysis process by deleting the *lacZ* gene in *E. coli* is an effective strategy to inactivate β-galactosidase activity [36]. Figure 4A shows a gradual decrease in the concentration of lactose during the prolonged fermentation time when the engineered strain EHD07 harboring pET-*nagA-glmM*, pRSF-*glmU*, and pRSF-*lgtA*(Cm^R^) grew in GlcNAc-defined medium I. Notably, at about 36 h of fermentation time, the lactose has been consumed by about 96.34%. The final concentration of accumulated LNT II in engineered EHD07 was about 0.20 g L^−1^. If *lacZ* is knocked out on the basis of the EHD07 strain, yielding EHD08 (EHD07Δ*lacZ*), lactose remains at a high residual level throughout the fermentation process, with a consumption rate of only 24.4% after fermentation for 72 h (Fig. 4B). The final titer of LNT II in engineered EHD08 was about 0.26 g L^−1^, with a gain of 30% compared to that of the engineered EHD07 strain. In contrast, the branched metabolic reaction of GlcNAc-6-P to ManNAc-6-P catalyzed by NanE may also decrease the metabolic flux of GlcNAc-6-P to GlcN-6-P (Fig. 1). Thus, we further measured the deletion effect of *nanE* on the production of LNT II using EHD08 as a starting strain, generating the engineered EHD24 strain (EHD08Δ*nanE*). As shown in Fig. 4C, the final concentration of LNT II reached 0.41 g L^−1^ at 72 h, showing 1.05- and 0.58-fold increases in the titer when compared to engineered EHD07 and EHD08 strains, respectively. This indicates that the combinatorial gene deletion of *lacZ* and *nanE* was more in favor of the production of LNT II than only the deletion of *lacZ* or no deletion of *lacZ* and *nanE*. Interestingly, the final dry cell weight (DCW) of EHD08 (Δ*lacZ*) and EHD24 (Δ*lacZ*Δ*nanE*) at 72 h were similar to EHD07 (control) (Fig. 4). Therefore, the constructed ELN (*E. coli* BL21(DE3)Δ*lacZ*Δ*nanE*) strain was used as the next starting strain for LNT II production.

### Optimization of gene expression strength for LNT II production

**Fig. 5:**
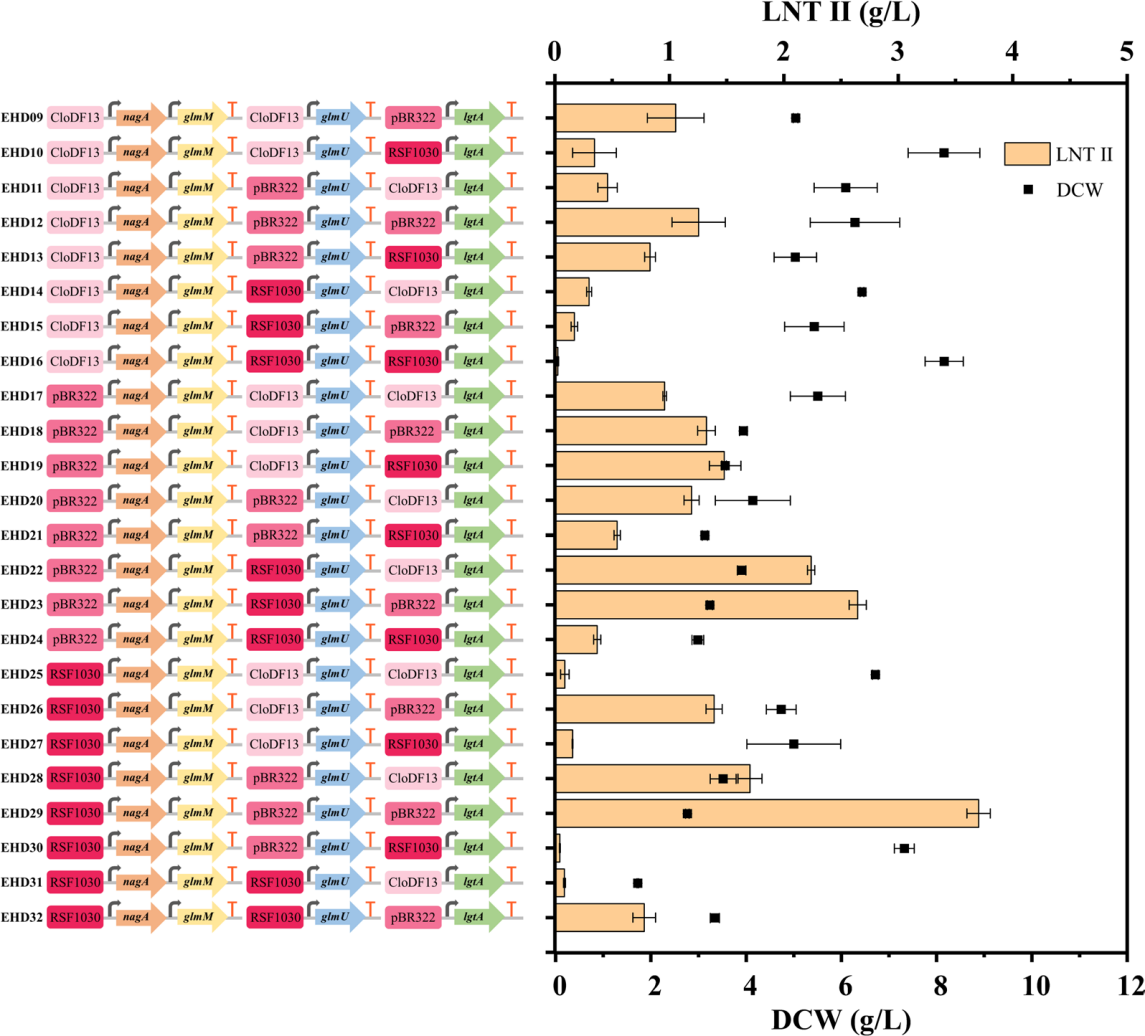
Fine-tuning of LNT II production via the optimization of gene (*nagA*, *glmM*, *glmU* and *lgtA*) expression strength using different plasmids including pCDFDuet-1, pETDuet-1 and pRSFDuet-1

As shown in Fig. 1, the biosynthesis of LNT II from GlcNAc-6-P required the simultaneous catalysis of four enzymes including NagA, GlmM, GlmU, and LgtA. The expression strength and quantity of these enzymes are very important for the metabolic balance between cell growth and product biosynthesis, because overexpression of protein may create unnatural burden to cells and change the global physiology, thus decreasing synthesis efficiency [37]. Currently, regulating gene expression strength by varying different plasmid copy numbers is an effective strategy to direct the metabolic flux to target products [38]. In this study, the vectors pCDFDuet-1 (20–40 copies), pETDuet-1 (~ 40 copies), and pRSFDuet-1 (~ 100 copies) were employed to construct a low-, medium-, and high-copy number, respectively, for the LNT II biosynthesis related enzymes, including NagA, GlmM, GlmU, and LgtA (Additional file 1: Table S1). Three different but compatible vectors were transformed in one strain to optimize the gene expression of these four enzymes. To enable the timely conversion and reinforcement of the metabolic flux of GlcNAc-6-P and GlcN-6-P to synthesize LNT II, and reduce the carbon flow into branched metabolism, *nagA* and *glmM* were inserted in one vector, considering that NagA and GlmM were responsible for catalyzing the first two-step reactions from GlcNAc-6-P to GlcN-1-P. In contrast, *glmU* and *lgtA* were individually inserted into another single vector to optimize their gene expression strength. To enable coexistence in the same engineered *E. coli* strain with pRSF-*nagA*-*glmM*, pET-*nagA-glmM,* and pCDF-*nagA-glmM* during the different vector combinations, the antibiotic resistance of the corresponding pRSF-*lgtA*, pET-*lgtA*, and pCDF-*lgtA*, pRSF-*glmU*, pET-*glmU*, and pCDF-*glmU* plasmids were changed to Cm. Through the orthogonal combinations using the three vectors expressing four enzymes (NagA, GlmM, GlmU, and LgtA), 24 engineered *E. coli* strains designated as EHD09 to EHD32 were generated in this study (Fig. 5 and Additional file 1: Table S1).

As shown in Fig. 5, the titers of LNT II and DCW of all the 24 engineered strains were measured and compared. The engineered strain EHD29 harboring pRSF-*nagA*-*glmM*, pET-*glmU,* and pET-*lgtA*(Cm^R^) with a high–medium–medium copy number combination yielded the highest LNT II titer of 3.70 g L^−1^, followed by EHD23 harboring pET-*nagA*-*glmM*, pRSF-*glmU,* and pET-*lgtA*(Cm^R^) with a medium–high–medium copy number combination producing 2.64 g L^−1^ of LNT II. By comparing the combination of plasmids harbored in EHD29 and EHD23, the overexpression of NagA and GlmM employing the higher copy number vector was more favorable for LNT II production than GlmU. Similar results were also found in other comparison groups between EHD26 (pRSF-*nagA-glmM*, pCDF-*glmU* and pET-*lgtA*, 1.39 g L^−1^) and EHD15 (pCDF-*nagA-glmM*, pRSF-*glmU* and pET-*lgtA*, 0.30 g L^−1^), EHD18 (pET-*nagA-glmM*, pCDF*-glmU* and pET-*lgtA* (Cm^R^), 1.32 g L^−1^) and EHD12 (pCDF-*nagA-glmM*, pET*-glmU* and pET-*lgtA* (Cm^R^), 1.25 g L^−1^), EHD19 (pET-*nagA-glmM*, pCDF-*glmU* and pRSF-*lgtA*, 1.48 g L^−1^) and EHD13 (pCDF-*nagA-glmM*, pET-*glmU* and pRSF-*lgtA*, 0.83 g L^−1^). A possible explanation is that overexpression of NagA and GlmM accelerates the conversion of GlcNAc-6-P to GlcN-1-P, thereby reducing the waste of branched carbon flow to Fru-6-P caused by NagB (Fig. 1). Interestingly, the overexpression of LgtA using a medium copy number pETDuet-1 vector is more beneficial for the production of LNT II than the expression by a high-copy number pRSFDuet-1 or low-copy number pCDFDuet-1vector, such as the comparison of combinatorial engineered strain EHD11/12/13, EHD14/15/16, EHD22/23/24, EHD25/26/27, and EHD28/29/30 (Fig. 5). The efficient and soluble expression of LgtA is another important factor for the biosynthesis of LNT II, as the heterologously expressed LgtA in *E. coli* tends to form inclusion bodies. Zhu et al. indicated that LgtA harbored by the pETDuet-1 vector presented relatively higher soluble expression level in engineered *E. coli* than the pRSFDuet-1 and pCDFDuet-1 vectors, according to the western blot results [13]. Our experimental results are consistent with their conclusions. Furthermore, the lowest titer of LNT II (0.025 g L^−1^) occurred in the engineered EHD16 strain harboring pCDF-*nagA-glmM*, pRSF-*glmU,* and pRSF-*lgtA* (Cm^R^). This could be attributed to the lower overexpression of NagA and GlmM, which affects the conversion speed of GlcNAc-6-P to GlcN-1-P, and higher forming inclusion bodies of LgtA that are unfavorable to LNT II production.

### Production of LNT II in 3-L bioreactor

**Fig. 6:**
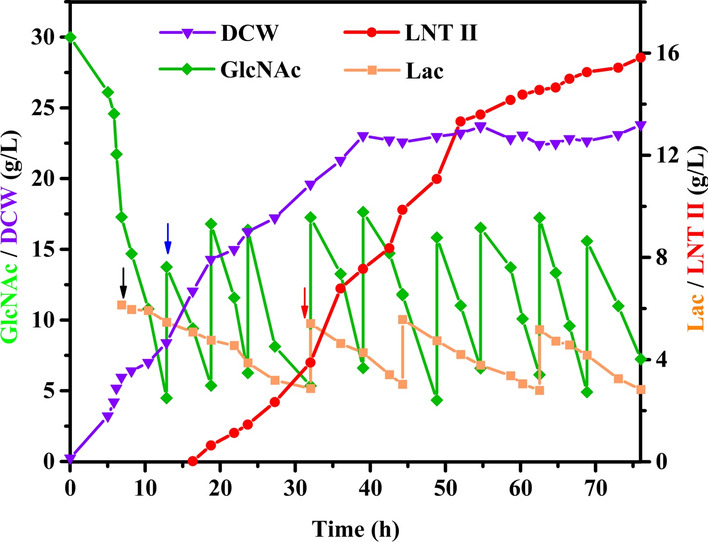
Time course curves of DCW of engineered *E. coli* and concentration of LNT II, GlcNAc and lactose during the fermentation of engineered *E. coli* in 3-L bioreactor

The large-scale production of LNT II was conducted in a 3-L bioreactor containing 1 L of GlcNAc-defined medium II using EHD29, namely *E. coli* BL21(DE3)Δ*lacZ*Δ*nanE* harboring pRSF-*nagA*-*glmM*, pET-*glmU,* and pET-*lgtA*(Cm^R^). During the fermentation process for LNT II production, GlcNAc not only served as a sole carbon source for cell growth, but also provided a precursor skeleton for the synthesis of LNT II, playing a dual role in regulating the cell growth and product synthesis. Therefore, an intermittent feeding strategy was adopted using GlcNAc as the carbon source and donor, and lactose as the acceptor to achieve efficient production of LNT II. Figure 6 shows that GlcNAc was consumed rapidly after about 12.8 h of fermentation, resulting in a residue concentration below 4.5 g L^−1^. Simultaneously, the DCW reached 8.4 g L^−1^. Subsequently, GlcNAc was first supplemented to the system to meet the requirement for cell growth. In contrast, IPTG was added in the bioreactor for the induction of T7 promoter-mediated gene expression after growing at about 6.8 h of the strain. The concentration of LNT II could be first detected by HPLC with a titer of 0.61 g L^−1^ after approximately 12 h of induction. Moreover, it could be seen that the consumption rate of GlcNAc was higher than that of lactose throughout the fermentation process. This is because lactose is not hydrolyzed to Glc and Gal due to knock-out of the *lacZ*, and the consumption of lactose only acts as an acceptor for LNT II synthesis, whereas the decreased GlcNAc is not only responsible for cell growth as carbon source, but also for LNT II synthesis as a backbone donor. Finally, the maximum titer of LNT II reached 15.8 g L^−1^ after fermentation for 76 h, which was 4.27-fold to that of the shake flask fermentation. The highest DCW of EHD29 reached 23.8 g L^−1^, which was 8.6-fold to that of the shake flask fermentation. These results demonstrate that the engineered *E. coli* cell factory can be used to synthesize LNT II from GlcNAc feedstock, a monomer of chitin.

Numerous studies have been conducted on the production of LNT II from chitin biomass or its hydrolysates (Table 1). Using a novel β-*N*-acetylhexosaminidase expressed by *Pichia pastoris*, 8.6 g L^−1^ of LNT II was produced from the reaction system composed of (GlcNAc)_2_ (glycosyl donor), one of chitin hydrolysates and β-lactose (glycosyl acceptor) [8]. However, a large amount of GlcNAc monomer remains in the reaction system due to the transglycosylation of β-*N*-acetylhexosaminidase towards (GlcNAc)_2_ and the hydrolysis of chitin by PbChi70, which is a potential issue to be considered. Further, LNT II could be in vitro produced from GlcNAc feedstock using the OPME method [9]. However, this production strategy is only achieved at the level of laboratory research, and certain challenges remain for large scale-up production in industry. Overall, to the best of our knowledge, this is the first report on producing LNT II through the fermentation strategy from GlcNAc feedstock using an engineered *E. coli* cell factory, providing a novel platform for the use of GlcNAc, a monomer of chitin, to produce high value-added products.

## Conclusions

In this study, the *E. coli* cell factory was first constructed for LNT II biosynthesis based on GlcNAc feedstock after introducing heterologous NmlgtA. The combinatorial deletion of the bypass pathway-related genes *lacZ* and *nanE* was beneficial for strengthening the production of LNT II, showing a 1.05-fold increase in titer when comparing with original strain. The overexpression of NagA and GlmM employing higher copy number vector was more favorable for LNT II production than GlmU and LgtA. The titer of LNT II was increased from 0.12 to 3.70 g L^−1^ step by step in shake flask cultivation. Finally, a titer of 15.8 g L^−1^ of LNT II was achieved by *E. coli* BL21(DE3)Δ*lacZ*Δ*nanE* harboring pRSF-*nagA*-*glmM*, pET-*glmU* and pET-*lgtA*(Cm^R^) in 3-L bioreactor. All in all, our study demonstrated that the engineered *E. coli* cell factory could be used for producing LNT II from GlcNAc feedstock, providing a new utilization for GlcNAc to produce high value-added products.

## Materials and methods

### Bacterial strains and plasmids

The Additional file 1: Table S1 lists all strains and plasmids used in this study. *E. coli* DH5α was used as a host strain for cloning and constructing plasmids. *E. coli* BL21(DE3) provided genomic templates for obtaining target genes *nagA*, *glmM,* and *glmU*. Plasmids pCDFDuet-1, pETDuet-1, pRSFDuet-1 (Novagen) were used for gene cloning and expression. pCas (Addgene, #62225) and pTargetF (Addgene, #62226) were purchased from Addgene and used for CRISPR–Cas9-mediated genome editing. Molecular kits for gel recovery and plasmid extraction were purchased from Sangon Biotech (Shanghai, China).

All primers used in this study are listed in Additional file 1: Table S2 and synthesized by GENEWIZ (Suzhou, China). The *lgtA* from *Neisseria meningitidis* was codon-optimized by Sangon Biotech (the sequence is shown in Additional file 1: Table S3) and constructed in three plasmid vectors: pRSFDuet-1, pETDuet-1, and pCDFDuet-1 to obtain plasmids: pRSF-*lgtA*, pET-*lgtA,* and pCDF-*lgtA*, respectively [16]. Due to the incompatibility of the same plasmids, antibiotic resistance of corresponding vectors was replaced by chloramphenicol (Cm), generating pCDFDuet-1(Cm^R^), pETDuet-1(Cm^R^), and pRSFDuet-1(Cm^R^). To modulate the gene expression strength for LNT II synthesis, plasmids with different copy numbers (pCDF-*nagA*-*glmM*, pET-*nagA*-*glmM*, pRSF-*nagA*-*glmM*, pCDF-*glmU*, pET-*glmU*, pRSF-*glmU*, pCDF-*glmU*(Cm^R^), pET-*glmU*(Cm^R^), pRSF-*glmU*(Cm^R^), pCDF-*lgtA*, pET-*lgtA*, pRSF-*lgtA*, pCDF-*lgtA*(Cm^R^), pET-*lgtA*(Cm^R^), pRSF-*lgtA*(Cm^R^)) were used to optimize the expression of these genes. All plasmids in this study were constructed by the Gibson assembly method [39] (Samuel Miller Lab, UW, Seattle).

### Medium, culture conditions, and shake flask fermentation

The shake flask fermentation medium called GlcNAc-defined medium I was prepared and slightly modified from previous studies [40]. The GlcNAc-defined medium I was composed of 20 g L^−1^ GlcNAc, 10 g L^−1^ yeast extract, 13.5 g L^−1^ KH_2_PO_4_, 4.0 g L^−1^ (NH_4_)_2_HPO_4_, 1.7 g L^−1^ citric acid, 1.4 g L^−1^ MgSO_4_·7H_2_O, 4.5 mg L^−1^ thiamine, and 10 mL L^−1^ trace element solution. The trace element solution was composed of 10 g L^−1^ FeSO_4_·7H_2_O, 2.2 g L^−1^ ZnSO_4_·7H_2_O, 1.0 g L^−1^ CuSO_4_·5H_2_O, 0.38 g L^−1^ MnSO_4_·H_2_O, 0.02 g L^−1^ Na_2_B_4_O_7_·10H_2_O, 0.1 g L^−1^ (NH_4_)_6_ Mo_7_O_24_, and 2.0 g L^−1^ CaCl_2_. For the fed-batch fermentation medium referred to as GlcNAc-defined medium II, the components were the same as GlcNAc-defined medium I, except that the initial concentration of GlcNAc was adjusted to 30 g L^−1^. The initial pH of all media was adjusted to 6.8 before autoclaving at 115 °C for 30 min. If necessary, the corresponding antibiotics, including 100 μg mL^−1^ ampicillin (Amp), 25 μg mL^−1^ Cm, 30 μg mL^−1^ kanamycin (Kan), and 50 μg mL^−1^ streptomycin (Sm) were added in the fermentation medium based on the characteristics of vectors.

The shake flask fermentation was performed as follows. A single colony was inoculated into test tubes containing 4 mL of fresh Luria–Bertani (LB) medium containing 10 g L^−1^ tryptone, 5 g L^−1^ yeast extract, and 10 g L^−1^ NaCl, and cultivated at 37 °C, 200 rpm overnight. Subsequently, 200 μL of the cultures were inoculated into 250-mL shake flasks containing 20 mL of fresh GlcNAc-defined medium I, and continued to be cultivated at 37 °C and 200 rpm. When the optical density (OD_600nm_) reached 0.6–0.8, a final concentration of 1 mM isopropyl-β-d-thiogalactopyranoside (IPTG) and 5 g L^−1^ β-lactose were added in the fermentation medium, and further incubated at 25 °C, 200 rpm for LNT II production until the total fermentation time reached 72 h.

### CRISPR/Cas9-mediated gene knock-out of *lacZ* and *nanE*

The plasmid harboring the *Cas9* gene (pCas) was electroporated into the host, and then cultivated in LB medium containing 30 μg mL^−1^ Kan. Simultaneously, 30 mM l-arabinose was added to induce the expression of λ-red recombinase genes (*gam*, *bet* and *exo*) that could help increase the efficiency of homologous recombination. After cultivation at 30 °C for 3 h, the cells were harvested to prepare competent cells. The N_20_ upstream of single-guide RNA (sgRNA) of *lacZ* and *nanE* were designed via the online website http://chopchop.cbu.uib.no/, and subsequently these sequences were inserted into the pTargetF plasmid by reverse PCR using pTargetF plasmid as template, yielding pTargetF-sg-*lacZ* and pTargetF-sg-*nanE*, respectively. The upstream (500 bp) and downstream (500 bp) of the target gene locus (*lacZ* and *nanE*) were amplified from the *E. coli* BL21(DE3) genome, respectively, and then further fused together by overlapping PCR and inserted to the pTargetF-sg-*lacZ* and pTargetF-sg-*nanE* using the Gibson assembly method, respectively, yielding pTargetT-Δl*acZ* and pTargetT-Δ*nanE* plasmids. The constructed pTargetT-Δl*acZ* and pTargetT-Δ*nanE* were further transformed into competent cells harboring pCas by electroporation. After cultivation at 30 °C for 1 h to recover the competent cells using LB medium, the cells were spread on the LB agar plate containing Kan and Sm overnight at 30 °C. The pTargetT plasmid of the identified colonies was cured by IPTG induction, and the pCas plasmid was killed by incubation at 42 °C overnight.

### Microbial production of LNT II in 3‑L bioreactor

The fed-batch fermentation was carried out in a 3-L bioreactor (Eppendorf, Juelich, Germany) containing 1 L of GlcNAc-defined medium II. The detailed operation steps were performed as follows. A single colony was inoculated into test tubes containing 4 mL of fresh LB medium, and cultivated at 37 °C and 200 rpm overnight. Then, 1 mL of cultures was inoculated into 500-mL shake flasks containing 100 mL of GlcNAc-defined medium I, and cultivated at 37 °C and 200 rpm to obtain primary seed cultures until the OD_600nm_ reached 0.6–0.8. The preparation process of secondary seed cultures was the same as that of primary seed cultures. Subsequently, 100 mL of secondary seed cultures were all transferred into the 3-L bioreactor. The airflow rate was set at 1.5–2.0 vvm. The dissolved oxygen was dynamically controlled by about 30% by automatically adjusting the agitation speed of 300–1000 rpm. The pH of the fermentation broth was maintained at 6.9 ± 0.2 by addition of NaOH (6 M) and H_2_SO_4_ (3 M) solution under the pH–stat mode. When the OD_600nm_ reached 17, the temperature was shifted to 20 °C for 20 min, and IPTG and lactose were injected to the bioreactor at a final concentration of 0.2 mM and 5 g L^−1^, respectively. During the entire cultivation, when the residue concentration of GlcNAc was below 5 g L^−1^, a storage solution containing 300 g L^−1^ of GlcNAc, 20 g L^−1^ of MgSO_4_.7H_2_O, and 0.2 g L^−1^ of thiamine were injected to make the final concentration of GlcNAc reach about 15 g L^−1^. When the residue concentration of β*-*lactose was below 3 g L^−1^, a storage solution containing 200 g L^−1^ of β*-*lactose was also injected to make the final concentration of β*-*lactose reach about 5 g L^−1^.

### Analytical methods

The OD_600nm_ was measured by a spectrophotometer, and the dry cell weight (DCW) was calculated from OD_600nm_ data based on the formula (1 OD_600nm_ ≈ 0.35 g L^−1^) [39]. The cells were collected from the culture broth by centrifugation at 12,000 rpm for 5 min. High-performance liquid chromatography (HPLC) (Waters e2695, Milford, MA, USA) equipped with the Rezex ROA-Organic Acid H^+^ (8%) column (Phenomenex Inc., Torrance, CA, USA) was used to detect the content of LNT II, β*-*lactose, and GlcNAc in the culture broth. A total of 5 mM H_2_SO_4_ was used as the mobile phase and eluted at a flow rate of 0.6 mL min^−1^ under a column temperature of 60 °C. An ultra-performance liquid chromatography–mass spectrometry (UPLC–MS) system was used to identify the LNT II product in the culture broth. The WATERS MALDI SYNAPT Q-TOF MS (Milford, MA, USA) equipped with a WATERS ACQUITY UPLC chromatograph and a BEH AMIDE analysis column (2.1 mm × 100 mm, 1.7 μm) was used to analyze the standards and samples under the positive ion mode using the following conditions: capillary, 3.5 kV; cone, 20 V; source block temperature, 100 °C; desolvation temperature, 400 °C; desolvation gas flow, 700 L h^−1^; cone gas flow, 50 L h^−1^; collision energy, 6 eV; mass range (m/z), 50–1500; detector voltage, 2100 Volts. The column was eluted with the mobile phase solvent A (80% (v/v) acetonitrile and 0.1% (v/v) NH_3_·H_2_O) and solvent B (30% (v/v) acetonitrile and 0.1% (v/v) NH_3_·H_2_O) at a flow rate of 0.3 mL min^−1^ at 45 ℃. The time elution procedure was as follows: 0 min, (100% A); 0.1–6 min, (60% A and 40% B); 6–6.1 min, (100% A).

## Supplementary Information

**Additional file 1: Table S1.** Engineered *E. coli* strains and constructed plasmids used in this study. **Table S2.** Primers used in this study. **Table S3.** Sequence of codon-optimized *lgtA* gene.

## Data Availability

All data generated or analyzed during this study are included in this published article and its additional files.

## Abbreviations

LNT II: Lacto-*N*-triose II
GlcNAc: *N*-Acetylglucosamine
HMOs: Human milk oligosaccharides
Glc: Glucose
Gal: Galactose
l-Fuc: Fucose
SA: Sialic acid
LNnT: Lacto-*N*-neotetraose
LNT: Lacto-*N*-tetraose
LgtA: β-1,3-Acetylglucosaminyltransferase
GlmS: Glucosamine-6-phosphate synthase
GlmM: Glucosamine synthase
GlmU: UDP-*N*-acetylglucosamine pyrophosphorylase
Fru-6-P: Fructose-6-phosphate
GlcN-6-P: Glucosamine-6-phosphate
GlcNAc-6-P: *N*-Acetylglucosamine-6-phosphate
Lac: Lactose
DCW: Dry cell weight
HPLC: High-performance liquid chromatography

## References

[1] Bode L (2012). Human milk oligosaccharides: every baby needs a sugar mama. Glycobiology.

[2] Zhu Y, Wan L, Li W, Ni D, Zhang W, Yan X, Mu W (2020). Recent advances on 2′-fucosyllactose: physiological properties, applications, and production approaches. Crit Rev Food Sci Nutr.

[3] Stuebe A (2009). The risks of not breastfeeding for mothers and infants. Rev Obstet Gynecol.

[4] Lucas A, Cole TJ (1990). Breast milk and neonatal necrotising enterocolitis. Lancet.

[5] Zhu Y, Luo G, Wan L, Meng J, Lee SY, Mu W (2021). Physiological effects, biosynthesis, and derivatization of key human milk tetrasaccharides, lacto-N-tetraose and lacto-N-neotetraose. Crit Rev Biotechnol.

[6] Faijes M, Castejón-Vilatersana M, Val-Cid C, Planas A (2019). Enzymatic and cell factory approaches to the production of human milk oligosaccharides. Biotechnol Adv.

[7] Chen X, Baker DC, Horton D (2015). Human milk oligosaccharides (HMOs): structure, function, and enzyme-catalyzed synthesis. Advances in carbohydrate chemistry and biochemistry.

[8] Liu Y, Yan Q, Ma J, Yang S, Li T, Jiang Z (2020). Production of lacto-N-triose II and lacto-N-neotetraose from chitin by a novel β-N-acetylhexosaminidase expressed in Pichia pastoris. ACS Sustain Chem Eng.

[9] McArthur JB, Yu H, Chen X (2019). A bacterial beta1-3-galactosyltransferase enables multigram-scale synthesis of human milk lacto-N-tetraose (LNT) and its fucosides. ACS Catal.

[10] Cheng L, Kiewiet MBG, Groeneveld A, Nauta A, de Vos P (2019). Human milk oligosaccharides and its acid hydrolysate LNT2 show immunomodulatory effects via TLRs in a dose and structure-dependent way. J Funct Foods.

[11] Weichert S, Jennewein S, Hufner E, Weiss C, Borkowski J, Putze J (2013). Bioengineered 2'-fucosyllactose and 3-fucosyllactose inhibit the adhesion of Pseudomonas aeruginosa and enteric pathogens to human intestinal and respiratory cell lines. Nutr Res.

[12] Nyffenegger C, Nordvang RT, Zeuner B, Lezyk M, Difilippo E, Logtenberg MJ (2015). Backbone structures in human milk oligosaccharides: trans-glycosylation by metagenomic β-N-acetylhexosaminidases. Appl Microbiol Biotechnol.

[13] Luo G, Zhu Y, Meng J, Wan L, Zhang W, Mu W (2021). A novel β-1,4-galactosyltransferase from *Histophilus somni* enables efficient biosynthesis of lacto-N-neotetraose via both enzymatic and cell factory approaches. J Agric Food Chem.

[14] Liu YH, Wang L, Huang P, Jiang ZQ, Yan QJ, Yang SQ (2020). Efficient sequential synthesis of lacto-N-triose II and lacto-N-neotetraose by a novel β-N-acetylhexosaminidase from *Tyzzerella nexilis*. Food Chem.

[15] Baumgartner F, Conrad J, Sprenger GA, Albermann C (2014). Synthesis of the human milk oligosaccharide lacto-N-tetraose in metabolically engineered, plasmid-free *E. coli*. ChemBioChem.

[16] Zhu Y, Wan L, Meng J, Luo G, Chen G, Wu H (2021). Metabolic engineering of *Escherichia coli* for lacto-N-triose II production with high productivity. J Agric Food Chem.

[17] Bych K, Miks MH, Johanson T, Hederos MJ, Vigsnaes LK, Becker P (2019). Production of HMOs using microbial hosts—from cell engineering to large scale production. Curr Opin Biotechnol.

[18] Sun W, Ji W, Li N, Tong P, Cheng J, He Y (2013). Construction and expression of a polycistronic plasmid encoding N-acetylglucosamine 2-epimerase and N-acetylneuraminic acid lyase simultaneously for production of N-acetylneuraminic acid. Bioresour Technol.

[19] Tang M, Zhou W, Liu Y, Yan J, Gong Z (2018). A two-stage process facilitating microbial lipid production from N-acetylglucosamine by *Cryptococcus curvatus* cultured under non-sterile conditions. Bioresour Technol.

[20] Tang M, Wang Y, Zhou W, Yang M, Liu Y, Gong Z (2020). Efficient conversion of chitin-derived carbon sources into microbial lipid by the oleaginous yeast *Cutaneotrichosporon oleaginosum*. Bioresour Technol.

[21] Zhang A, Wei G, Mo X, Zhou N, Chen K, Ouyang P (2018). Enzymatic hydrolysis of chitin pretreated by bacterial fermentation to obtain pure N-acetyl-D-glucosamine. Green Chem.

[22] Wei G, Zhang A, Chen K, Ouyang P (2017). Enzymatic production of N-acetyl-D-glucosamine from crayfish shell wastes pretreated via high pressure homogenization. Carbohydr Polym.

[23] Villa-Lerma G, Gonzalez-Marquez H, Gimeno M, Lopez-Luna A, Barzana E, Shirai K (2013). Ultrasonication and steam-explosion as chitin pretreatments for chitin oligosaccharide production by chitinases of *Lecanicillium lecanii*. Bioresour Technol.

[24] Sivaramakrishna D, Bhuvanachandra B, Nadendla SR, Podile AR (2020). Efficient conversion of alpha-chitin by multi-modular chitinase from *Chitiniphilus shinanonensis* with KOH and KOH-urea pretreatment. Carbohyd Polym.

[25] Roy I, Mondal K, Gupta MN (2003). Accelerating enzymatic hydrolysis of chitin by microwave pretreatment. Biotechnol Prog.

[26] Hou F, Ma X, Fan L, Wang D, Ding T, Ye X (2020). Enhancement of chitin suspension hydrolysis by a combination of ultrasound and chitinase. Carbohyd Polym.

[27] Wang YT, Wu PL (2020). Gene cloning, characterization, and molecular simulations of a novel recombinant chitinase from Chitinibacter Tainanensis CT01 appropriate for chitin enzymatic hydrolysis. Polymers (Basel).

[28] Nguyen-Thi N, Doucet N (2016). Combining chitinase C and N-acetylhexosaminidase from Streptomyces coelicolor A3(2) provides an efficient way to synthesize N-acetylglucosamine from crystalline chitin. J Biotechnol.

[29] Ola B, van Irma D, Thomas N, van den Eijnden DH (1999). High-level expression of the *Neisseria meningitidis* lgtA gene in *Escherichia coli* and characterization of the encoded N-acetylglucosaminyltransferase as a useful catalyst in the synthesis of GlcNAcβ1→3Gal and GalNAcβ1→3Gal linkages. Glycobiology.

[30] Chen Y, Thon V, Li Y, Yu H, Ding L, Lau K (2011). One-pot three-enzyme synthesis of UDP-GlcNAc derivatives. Chem Commun.

[31] Badet B, Vermoote P, Le Goffic F (1988). Glucosamine synthetase from *Escherichia coli*: kinetic mechanism and inhibition by N3-fumaroyl-L-2,3-diaminopropionic derivatives. Biochemistry.

[32] Broschat KO, Gorka C, Page JD, Martin-Berger CL, Davies MS, Huang H (2002). Kinetic characterization of human glutamine-fructose-6-phosphate amidotransferase I: Potent feedback inhibition by glucosamine 6-phosphate. J Biol Chem.

[33] Deng MD, Grund AD, Wassink SL, Peng SS, Nielsen KL, Huckins BD (2006). Directed evolution and characterization of *Escherichia coli* glucosamine synthase. Biochimie.

[34] Jiang Y, Chen B, Duan C, Sun B, Yang J, Yang S (2015). Multigene editing in the *Escherichia coli* genome via the CRISPR-Cas9 system. Appl Environ Microbiol.

[35] Li W, Zhu Y, Wan L, Guang C, Mu W (2021). Pathway optimization of 2′-fucosyllactose production in engineered *Escherichia coli*. J Agric Food Chem.

[36] Priem B, Gilbert M, Wakarchuk WW, Heyraud A, Samain E (2002). A new fermentation process allows large-scale production of human milk oligosaccharides by metabolically engineered bacteria. Glycobiology.

[37] Ceroni F, Boo A, Furini S, Gorochowski TE, Borkowski O, Ladak YN (2018). Burden-driven feedback control of gene expression. Nat Methods.

[38] Wan L, Zhu Y, Li W, Zhang W, Mu W (2020). Combinatorial modular pathway engineering for guanosine 5'-diphosphate-L-fucose production in recombinant *Escherichia coli*. J Agric Food Chem.

[39] Gibson DG, Young L, Chuang RY, Venter JC, Hutchison CA, Smith HO (2009). Enzymatic assembly of DNA molecules up to several hundred kilobases. Nat Methods.

[40] Pang Q, Han H, Liu X, Wang Z, Liang Q, Hou J (2020). In vivo evolutionary engineering of riboswitch with high-threshold for N-acetylneuraminic acid production. Metab Eng.

