# Supplementary material for: Engineering Escherichia coli for highly efficient production of lacto-N-triose II from N-acetylglucosamine, the monomer of chitin
Source: Biotechnol Biofuels. 2021 Oct 8;14:198. doi: 10.1186/s13068-021-02050-5 (PMC8501739; doi:10.1186/s13068-021-02050-5)
Supplement: Supplementary file 1 — Additional file 1: Table S1. Engineered E. coli strains and constructed plasmids used in this study. Table S2. Primers used in this study. Table S3. Sequence of codon-optimized lgtA gene. [file 13068_2021_2050_MOESM1_ESM.docx]

**Table S1 *E. coli* strains and plasmids used in this study**

| **Names** | **Relevant genotype and description** | **References** |
| --- | --- | --- |
| **Strains** |  |  |
| DH5α | F^−^ *endA1 glnV44 thi-1 recA1 relA1 gyrA96 deoR nupG* Φ80d*lacZ*ΔM15 Δ(*lacZYA-argF*) *U169 hsdR17* (r_K_^−^m_K_^＋^) *λ*^−^ | type strain |
| BL21(DE3) | F^−^ *ompT hsdSB* (r_B_^−^m_B_^−^) *gal dcm rne131 λ* (DE3) | type strain |
| EL | *E. coli* BL21(DE3) Δ*lacZ* | this study |
| ELN | *E. coli* BL21(DE3) Δ*lacZ*Δ*nanE* | this study |
| EHD01 | *E. coli* BL21(DE3) | this study |
| EHD02 | EHD01 harboring plasmids pET-*lgtA* | this study |
| EHD03 | EHD01 harboring plasmids pET-*lgtA* and pCDF-*glmU* | this study |
| EHD04 | EHD01 harboring plasmids pET-*lgtA*, pCDF-*glmU* and pRSF-*glmM* | this study |
| EHD05 | EHD01 harboring plasmids pET-*lgtA*, pCDF-*glmU* and pRSF-*nagA*-*glmM* | this study |
| EHD06 | EHD01 harboring plasmids pCDF-*glmU* and pRSF-*nagA*-*glmM* | this study |
| EHD07 | EHD01 harboring plasmids pET-*nagA-glmM*, pRSF-*glmU* and pRSF-*lgtA*(Cm^R^) | this study |
| EHD08 | EL harboring plasmids pET-*nagA-glmM*, pRSF-*glmU* and pRSF-*lgtA*(Cm^R^) | this study |
| EHD24 | ELN harboring plasmids pET-*nagA-glmM*, pRSF-*glmU* and pRSF-*lgtA*(Cm^R^) | this study |
| EHD09 | ELN harboring plasmids pCDF-*nagA-glmM*, pCDF-*glmU* (Cm^R^) and pET-*lgtA* | this study |
| EHD10 | ELN harboring plasmids pCDF-*nagA-glmM*, pCDF-*glmU* (Cm^R^) and pRSF-*lgtA* | this study |
| EHD11 | ELN harboring plasmids pCDF-*nagA-glmM*, pET-*glmU* and pCDF-*lgtA* (Cm^R^) | this study |
| EHD12 | ELN harboring plasmids pCDF-*nagA-glmM*, pET*-glmU* and pET-*lgtA* (Cm^R^) | this study |
| EHD13 | ELN harboring plasmids pCDF-*nagA-glmM*, pET-*glmU* and pRSF-*lgtA* | this study |
| EHD14 | ELN harboring plasmids pCDF-*nagA-glmM*, pRSF-*glmU* and pCDF-*lgtA* (Cm^R^) | this study |
| EHD15 | ELN harboring plasmids pCDF-*nagA-glmM*, pRSF-*glmU* and pET-*lgtA* | this study |
| EHD16 | ELN harboring plasmids pCDF-*nagA-glmM*, pRSF-*glmU* and pRSF-*lgtA* (Cm^R^) | this study |
| EHD17 | ELN harboring plasmids pET-*nagA-glmM*, pCDF-*glmU* and pCDF-*lgtA*(Cm^R^) | this study |
| EHD18 | ELN harboring plasmids pET-*nagA-glmM*, pCDF-*glmU* and pET-*lgtA* (Cm^R^) | this study |
| EHD19 | ELN harboring plasmids pET-*nagA-glmM*, pCDF-*glmU* and pRSF-*lgtA* | this study |
| EHD20 | ELN harboring plasmids pET-*nagA-glmM*, pET-*glmU* (Cm^R^) and pCDF-*lgtA* | this study |
| EHD21 | ELN harboring plasmids pET-*nagA-glmM*, pET-*glmU* (Cm^R^) and pRSF-*lgtA* | this study |
| EHD22 | ELN harboring plasmids pET-*nagA-glmM*, pRSF-*glmU* and pCDF-*lgtA* | this study |
| EHD23 | ELN harboring plasmids pET-*nagA-glmM*, pRSF-*glmU* and pET-*lgtA* (Cm^R^) | this study |
| EHD24 | ELN harboring plasmids pET-*nagA-glmM*, pRSF-*glmU* and pRSF-*lgtA* (Cm^R^) | this study |
| EHD25 | ELN harboring plasmids pRSF-*nagA-glmM*, pCDF-*glmU* and pCDF-*lgtA* (Cm^R^) | this study |
| EHD26 | ELN harboring plasmids pRSF-*nagA-glmM*, pCDF-*glmU* and pET-*lgtA* | this study |
| EHD27 | ELN harboring plasmids pRSF*-nagA-glmM*, pCDF-*glmU* and pRSF-*lgtA* (Cm^R^) | this study |
| EHD28 | ELN harboring plasmids pRSF-*nagA-glmM*, pET-*glmU* and pCDF-*lgtA* | this study |
| EHD29 | ELN harboring plasmids pRSF-*nagA-glmM*, pET-*glmU* and pET-*lgtA* (Cm^R^) | this study |
| EHD30 | ELN harboring plasmids pRSF-*nagA-glmM*, pET-*glmU* and pRSF-*lgtA* (Cm^R^) | this study |
| EHD31 | ELN harboring plasmids pRSF-*nagA-glmM*, pRSF-*glmU* (Cm^R^) and pCDF-*lgtA* | this study |
| EHD32 | ELN harboring plasmids pRSF-*nagA-glmM*, pRSF-*glmU* (Cm^R^) and pET-*lgtA* | this study |
| **Plasmids** |  |  |
| pCDFDuet-1 | Two T7 promoters, CloDF13 replicon, Sm^R^, medium copy number, 20∼40 copies/cell | Novagen |
| pETDuet-1 | Two T7 promoters, pBR322 replicon, Amp^R^, medium copy number, ∼40 copies/cell | Novagen |
| pRSFDuet-1 | Two T7 promoters, RSF1030 replicon, Kan^R^, high copy number, ∼100 copies/cell | Novagen |
| pCDF-*nagA-glmM* | pCDFDuet-1 + T7 promoter- *nagA*(MCS1) + T7 promoter-*glmM*(MCS2) | this study |
| pET-*nagA-glmM* | pETDuet-1 + T7 promoter- *nagA*(MCS1) + T7 promoter-*glmM*(MCS2) | this study |
| pRSF-*nagA-glmM* | pRSFDuet-1 + T7 promoter- *nagA*(MCS1) + T7 promoter-*glmM*(MCS2) | this study |
| pCDFDuet-1(Cm^R^) | Two T7 promoters, CloDF13 replicon, cm^R^, medium copy number, 20 ∼ 40 copies/cell | laboratory |
| pETDuet-1(Cm^R^) | Two T7 promoters, pBR322 replicon, cm^R^, medium copy number, ∼40 copies/cell | laboratory |
| pRSFDuet-1(Cm^R^) | Two T7 promoters, RSF1030 replicon, cm^R^, high copy number, ∼100 copies/cell | laboratory |
| pCDF-*glmU* | pCDFDuet-1 + T7 promoter- *glmU* (MCS1) | this study |
| pET-*glmU* | pETDuet-1 + T7 promoter- *glmU* (MCS1) | this study |
| pRSF-*glmU* | pRSFDuet-1 + T7 promoter- *glmU* (MCS1) | this study |
| pCDF-*glmU*(Cm^R^) | pCDFDuet-1 (Cm^R^)+ T7 promoter- *glmU* (MCS1) | this study |
| pET-*glmU*(Cm^R^) | pETDuet-1 (Cm^R^) + T7 promoter- *glmU* (MCS1) | this study |
| pRSF-*glmU*(Cm^R^) | pRSFDuet-1 (Cm^R^) + T7 promoter- *glmU* (MCS1) | this study |
| pCDF-*lgtA* | pCDFDuet-1 + T7 promoter- *lgtA* (MCS1) | this study |
| pET-*lgtA* | pETDuet-1 + T7 promoter- *lgtA* (MCS1) | this study |
| pRSF-*lgtA* | pRSFDuet-1 + T7 promoter- *lgtA* (MCS1) | this study |
| pCDF-*lgtA*(Cm^R^) | pCDFDuet-1 (Cm^R^)+ T7 promoter- *lgtA* (MCS1) | this study |
| pET-*lgtA*(Cm^R^) | pETDuet-1 (Cm^R^) + T7 promoter- *lgtA* (MCS1) | this study |
| pRSF-*lgtA*(Cm^R^) | pRSFDuet-1 (Cm^R^) + T7 promoter- *lgtA* (MCS1) | this study |
| pCas | *repA101* (Ts) *kan P_cas_-cas9 P_araB_-Red lacI^q^P_trc_-*sgRNA*-pMB1*, Kan^R^ | Addgene (#62225) |
| pTargetF | *pMB1 aadA* sgRNA | Addgene (#62226) |
| pTargetF-sg-*nanE* | *pMB1 aadA* sgRNA-*nanE* | this study |
| pTargetT-Δ*nanE* | *pMB1 aadA* sgRNA-*nanE* , consisted of sgRNA sequence, N20, and donor editing template DNA | this study |
| pTargetF-sg-*lacZ* | *pMB1 aadA* sgRNA-*lacZ* | this study |
| pTargetT-Δ*lacZ* | *pMB1 aadA* sgRNA-*lacZ* , consisted of sgRNA sequence, N20, and donor editing template DNA | this study |

**Table S2 Primers used in this study**

| **Primers** | **Sequences (5’-3’)** |
| --- | --- |
| **Gene knockout** |  |
| *lacZ*-up-F | TTGGCAACCGTGGCAGAAG |
| *lacZ-*up-R | GACTGGGAAAACCCTGGCCGGTCGCTACCATTACCAGTTG |
| *lacZ*-down-F | CAACTGGTAATGGTAGCGACCGGCCAGGGTTTTCCCAGTC |
| *lacZ*-down-R | GGTAGTGGGATACGACGATACC |
| pTargetF-*lacZ*-F | CGGGTGAACTGATCGCGCAGGTTTTAGAGCTAGAAATAGCAAGTT |
| pTargetF-*lacZ*-R | CTGCGCGATCAGTTCACCCGACTAGTATTATACCTAGGACTGAGCTAG |
| *nanE*-up-R | ataaacccgaaaacgcgtcttgaacacatttg |
| *nanE*-down-F | aaatgtgttcaagacgcgttttcgggtttatcgag |
| *nanE*-down-R | ttaccaaaactgattggcggttatttcgataccg |
| pTargetF-*nanE*-F | AAATCTGCAAGCCACGCGTGgttttagagctagaaatagcaagttaaaataag |
| pTargetF-*nanE*-R | CACGCGTGGCTTGCAGATTTactagtattatacctaggactgagctag |
| pTargetT-link-*nanE*-F | ttaatggcaatggtcggcaaattggtaagttgttcc |
| pTargetT-link-*nanE*-R | ataaacccgaaaacgcgtcttgaacacatttg |
| pTargetT-link-*nanE*-V-F | aaatgtgttcaagacgcgttttcgggtttatcgag |
| pTargetT-link-*nanE*-V-R | gccgaccattgccattaagaattcaaaaaaagcacc |
| **Plasmid construction** |  |
| pCDF-*nagA*-F | CTTTAATAAGGAGATATACCATGGGCatgtatgcattaacccagggccg |
| pCDF-*nagA*-V-R | catGCCCATGGTATATCTCCTTATTAAAGTTAAACAAAATTATTTCTAC |
| pCDF-*nagA*-V-F | gaggtcgtaactcaataaAGCAGCCATCACCATCATCACc |
| pCDF-*nagA*-R | GATGGCTGCTttattgagttacgacctcgttaccgttaacg |
| pCDF-*glmU*-F | GATATACCATGGGCatgttgaataatgctatgagcgtagtgatcc |
| pCDF-*glmU*-V-R | gcattattcaacatGCCCATGGTATATCTCCTTATTAAAGTTAAAC |
| pCDF-*glmU*-V-F | cgtccggtaaagaaaaagtgaAGCAGCCATCACCATCATC |
| pCDF-*glmU*-R | GGCTGCTtcactttttctttaccggacgacgc |
| pCDF-*nagA*-*glmM*-F | GTATAAGAAGGAGATATACATATGagtaatcgtaaatatttcggtaccgatggg |
| pCDF-*nagA*-*glmM*-V-R | tactCATATGTATATCTCCTTCTTATACTTAACTAATATACTAAGATGGGG |
| pCDF-*nagA*-*glmM*-V-F | gatgcagtaaaagccgtttaaGCAGATCTCAATTGGATATCGGCC |
| pCDF-*nagA*-*glmM*-R | GATCTGCttaaacggcttttactgcatcggc |
| PCDF-*lgtA*-F | gtttaactttaataaggagatataccATGggcCAGCCGCTG |
| pCDF-*lgtA*-V-R | CATggtatatctccttattaaagttaaacaaaattatttctacagggg |
| pCDF-*lgtA*-V-R | CATggtatatctccttattaaagttaaacaaaattatttctacagggg |
| pCDF-*lgtA*-V-F | CTGAAAAACCGTTAAgaattcgagctcggcgcg |
| pCDF-*lgtA*-R | cgagctcgaattcTTAACGGTTTTTCAGCAGACGGTGC |
| PET-*nagA*-F | CTTTAAGAAGGAGATATACCATGGGCatgtatgcattaacccagggccg |
| PET-*nagA*-V-R | catGCCCATGGTATATCTCCTTCTTAAAGTTAAACAAAATTATTTC |
| PET-*nagA*-V-F | ggtcgtaactcaataaAGCAGCCATCACCATCATCACC |
| PET-*nagA*-R | GGTGATGGCTGCTttattgagttacgacctcgttaccgttaacg |
| PET-*glmU*-F | GATATACCATGGGCatgttgaataatgctatgagcgtagtgatcc |
| PET-*glmU*-V-R | gcattattcaacatGCCCATGGTATATCTCCTTCTTAAAGTTAAAC |
| PET-*glmU*-V-F | ggtaaagaaaaagtgaAGCAGCCATCACCATCATCAC |
| PET-*glmU*-R | GGTGATGGCTGCTtcactttttctttaccggacgacgc |
| PET-*nagA*-*glmM*-F | aAGAAGGAGATATACATATGagtaatcgtaaatatttcggtaccgatgg |
| PET-*nagA*-*glmM*-V-R | cgattactCATATGTATATCTCCTTCTTATACTTAACTAATATACTAAGATGGGG |
| PET-*nagA*-*glmM*-V-F | gtaaaagccgtttaaGCAGATCTCAATTGGATATCGGCC |
| PET-*nagA*-*glmM*-R | CAATTGAGATCTGCttaaacggcttttactgcatcggc |
| PET-*lgtA*-F | ctttaagaaggagatataccATGggcCAGCCGCTG |
| PET-*lgtA*-V-R | CTGgccCATggtatatctccttcttaaagttaaacaaaattatttctagaggg |
| PET-*lgtA*-V-F | CGTCTGCTGAAAAACCGTTAAgaattcgagctcggcgcg |
| PET-*lgtA*-R | cgaattcTTAACGGTTTTTCAGCAGACGGTGC |
| PRSF-*nagA*-F | CTTTAATAAGGAGATATACCATGGGCatgtatgcattaacccagggccg |
| PRSF-*nagA*-V-R | catGCCCATGGTATATCTCCTTATTAAAGTTAAACAAAATTATTTC |
| PRSF-*nagA*-V-F | cgaggtcgtaactcaataaAGCAGCCATCACCATCATCAC |
| PRSF-*nagA*-R | GATGGCTGCTttattgagttacgacctcgttaccgttaacg |
| PRSF-*glmU*-F | GATATACCATGGGCatgttgaataatgctatgagcgtagtgatcc |
| PRSF-*glmU*-V-R | gcattattcaacatGCCCATGGTATATCTCCTTATTAAAGTTAAAC |
| PRSF-*glmU*-V-F | cgtccggtaaagaaaaagtgaAGCAGCCATCACCATCATC |
| PRSF-*glmU*-R | GGCTGCTtcactttttctttaccggacgacgc |
| pRSF-*nagA*-*glmM*-F | TAAGAAGGAGATATACATATGagtaatcgtaaatatttcggtaccgatggg |
| pRSF-*nagA*-*glmM*-V-R | cgattactCATATGTATATCTCCTTCTTATACTTAACTAATATACTAAGATGGGG |
| pRSF-*nagA*-*glmM*-V-F | gtaaaagccgtttaaGCAGATCTCAATTGGATATCGGCC |
| pRSF-*nagA*-*glmM*-R | CAATTGAGATCTGCttaaacggcttttactgcatcggc |
| pRSF-*lgtA*-F | gtttaactttaataaggagatataccATGggcCAGCCGCTG |
| pRSF-*lgtA*-V-R | CATggtatatctccttattaaagttaaacaaaattatttctacagggg |
| pRSF-*lgtA*-V-F | CGTCTGCTGAAAAACCGTTAAgaattcgagctcggcgcg |
| pRSF-*lgtA*-R | cgaattcTTAACGGTTTTTCAGCAGACGGTGC |

**Table S3** Sequences of codon-optimized *lgtA* gene

| Enzyme | Sequence (5’→3’) |
| --- | --- |
| LgtA | ATGGGCCAGCCGCTGGTTAGCGTTCTGATCTGCGCGTACAACGTTGAAAAATATTTCGCGCAGAGCCTGGCAGCTGTTGTTAACCAGACCTGGCGTAACCTGGACATTCTGATCGTTGATGATGGCTCTACCGATGGCACCCTGGCGATCGCGCAGCGTTTCCAGGAACAGGACGGTCGTATCCGTATTCTGGCGCAGCCGCGTAACTCTGGTCTGATTCCAAGCCTGAACATCGGCCTGGATGAACTGGCGAAAAGCGGCGGTGGTGGTGAATACATCGCGCGTACCGATGCGGATGATATCGCAGCTCCGGATTGGATTGAAAAAATCGTTGGTGAAATGGAAAAAGATCGTAGCATCATCGCAATGGGCGCTTGGCTGGAAGTGCTGTCCGAAGAAAAAGATGGCAACCGTCTGGCACGTCACCACGAACACGGTAAAATCTGGAAAAAACCGACCCGTCACGAAGACATCGCGGATTTCTTCCCATTCGGCAACCCGATTCACAACAACACCATGATCATGCGTCGTTCCGTGATCGATGGCGGCCTGCGTTACAACACCGAACGTGATTGGGCAGAAGACTATCAGTTCTGGTATGATGTTTCTAAACTGGGTCGTCTGGCGTACTACCCGGAAGCGCTGGTTAAATACCGTCTGCACGCTAACCAGGTTAGCTCCAAATATAGCATCCGCCAGCACGAAATCGCTCAGGGTATCCAGAAAACCGCACGTAACGATTTCCTGCAGTCTATGGGTTTCAAAACCCGTTTCGATAGCCTGGAATACCGTCAGATTAAAGCGGTTGCGTATGAACTGCTGGAAAAACACCTGCCGGAAGAAGATTTTGAACTGGCGCGTCGTTTCCTGTACCAGTGCTTCAAACGTACCGATACCCTGCCGGCGGGCGCTTGGCTGGATTTCGCGGCGGATGGCCGTATGCGTCGTCTGTTCACCCTGCGTCAGTACTTCGGTATCCTGCACCGTCTGCTGAAAAACCGTTAA |
